# Reversible Keap1 inhibitors are preferential pharmacological tools to modulate cellular mitophagy

**DOI:** 10.1038/s41598-017-07679-7

**Published:** 2017-09-04

**Authors:** Nikolaos D. Georgakopoulos, Michele Frison, Maria Soledad Alvarez, Hélène Bertrand, Geoff Wells, Michelangelo Campanella

**Affiliations:** 1 Department of Comparative Biomedical Sciences, The Royal Veterinary College, University of London, Royal College Street, NW1 0TU London, United Kingdom; 2 University College London Consortium for Mitochondrial Research, Gower Street, WC1 6BT London, United Kingdom; 3 UCL School of Pharmacy, 29/39 Brunswick Square, London, United Kingdom

## Abstract

Mitophagy orchestrates the autophagic degradation of dysfunctional mitochondria preventing their pathological accumulation and contributing to cellular homeostasis. We previously identified a novel chemical tool (hereafter referred to as PMI), which drives mitochondria into autophagy without collapsing their membrane potential (ΔΨ_m_). PMI is an inhibitor of the protein-protein interaction (PPI) between the transcription factor Nrf2 and its negative regulator, Keap1 and is able to up-regulate the expression of autophagy-associated proteins, including p62/SQSTM1. Here we show that PMI promotes mitochondrial respiration, leading to a superoxide-dependent activation of mitophagy. Structurally distinct Keap1-Nrf2 PPI inhibitors promote mitochondrial turnover, while covalent Keap1 modifiers, including sulforaphane (SFN) and dimethyl fumarate (DMF), are unable to induce a similar response. Additionally, we demonstrate that SFN reverses the effects of PMI in co-treated cells by reducing the accumulation of p62 in mitochondria and subsequently limiting their autophagic degradation. This study highlights the unique features of Keap1-Nrf2 PPI inhibitors as inducers of mitophagy and their potential as pharmacological agents for the treatment of pathological conditions characterized by impaired mitochondrial quality control.

## Introduction

Mitophagy is a highly selective degradation process that eliminates dysfunctional or superfluous mitochondria through the autophagic machinery^1^. It functions principally via the PINK1-PARK2 pathway, which is activated upon dissipation of the mitochondrial membrane potential (ΔΨ_m_)^2^. PINK1 (PTEN-induced putative kinase 1) and PARK2 act synergistically to flag depolarized mitochondria for degradation by decorating their surface with phospho-ubiquitin chains. This serves as a recognition signal for autophagy receptors, which in turn accumulate in mitochondria and facilitate their degradation by recruiting downstream components of the autophagic machinery^3^.

Impaired mitophagy leads to an accumulation of dysfunctional organelles and plays a pivotal role in the pathogenesis of cancer and neurodegenerative conditions, particularly Parkinson’s disease^4, 5^. Currently, the available means to modulate this process are limited to respiratory chain or phosphorylation inhibitors, and ionophores such as carbonyl cyanide-4-(trifluoromethoxy)phenylhydrazone (FCCP)^2, 6^. However, the therapeutic potential of such compounds is doubtful, as their mode of action depends largely on their ability to mediate mitochondrial-associated toxicity, thus highlighting the need for alternative chemical tools to modulate this process^7^.

The transcription factor Nrf2 (nuclear factor erythroid 2-related factor 2) regulates the expression of a battery of cytoprotective genes with Antioxidant Response Element (ARE) sequences in their promoter regions^8^. Amongst the gene products controlled by Nrf2, of particular relevance are proteins involved in quality control processes, such as PINK1^9^ and the autophagy receptors NDP52 (nuclear dot protein 52)^10^ and sequestosome1/p62^11^. We recently described the identification of a novel Nrf2 inducer (HB229/PMI) that increases the cellular levels of p62 by reversibly inhibiting the regulatory activity of Keap1 (Kelch-like ECH-associated protein 1), a redox sensitive protein that interacts with Nrf2 and mediates its degradation through the ubiquitin proteasome system^12^. PMI disrupts this protein-protein interaction (PPI), thereby blocking the ubiquitination of Nrf2 and promoting its nuclear accumulation^13^. The subsequent up-regulation of downstream gene products, including p62, initiates a mitophagic response without causing toxicity to the organelle or collapsing the ΔΨ_m_^12^. Moreover, the activity of PMI is retained in cells lacking a fully functional PINK1-PARK2 pathway, but not in Nrf2^−/−^ and p62^−/−^ mouse embryonic fibroblasts (MEFs).

Intriguingly, the prototype electrophilic Nrf2 inducer sulforaphane (SFN) does not mediate a similar effect on mitochondrial turnover, despite up-regulating p62. We hypothesized that the opposing effects of PMI and SFN might stem from their distinct mechanisms of Keap1 inhibition and selectivity profiles^13, 14^. In contrast to PMI, SFN halts the degradation of Nrf2 by covalently modifying reactive cysteine residues on Keap1 and subsequently, diminishing its ubiquitination facilitating activity^14^. However, due to its high reactivity, it is also capable of irreversibly modifying a wide range of redox sensitive proteins, which in turn may compromise the Nrf2-mediated effects on mitochondrial quality control^15–17^. Here we demonstrate that in contrast to PMI, SFN does not promote the recruitment of p62 to mitochondria, which in turn restricts mitophagy. These effects are predominant in co-treated cells and lead to an inhibition of the PMI-induced mitophagy, which appears to be dependent on mitochondrial superoxide metabolism. Interestingly, other chemotypes that inhibit the Keap1-Nrf2 PPI have similar effects to PMI and promote mitochondrial clearance, while covalent Keap1 inhibitors are unable to produce a similar response.

## Materials and Methods

### Chemicals

SFN, Carbonyl cyanide-*p*-trifluoromethoxyphenylhydrazone (FCCP), dimethylfumarate (DMF), curcumin and *tert*-Butylhydroquinone (TBHQ) were purchased from Sigma-Aldrich. PMI (compound **1**) and analogues (compounds **2**–**4**) were prepared as described previously^13^. Compounds **5** and **6** were synthesized starting from 1-nitronaphthalene using adaptations of the published procedures^18, 19^.

### Cell culture and transfections

Mouse embryonic fibroblasts (MEF), HeLa and SH-SY5Y (SHY) cells were cultured in Dulbecco’s modified Eagle medium (DMEM; Life Technologies, 41966-052) supplemented with 10% heat-inactivated fetal bovine serum (Life Technologies, 10082-147), 100 units/mL penicillin, and 100 mg/mL streptomycin (Life Technologies, 15140-122) at 37 °C under humidified conditions and 5% CO_2_. Cells were plated on glass coverslips at a 10% confluence and transiently transfected the following day using a standard Ca^2+^ phosphate method^20^. Cells were treated as described in figure legends 36 hours post-transfection. Experiments, unless otherwise indicated, were performed in Dulbecco’s modified Eagle medium supplemented with 10% fetal bovine serum or in the following recording medium (RM): 125 mM NaCl, 5 mM KCl, 1 mM NaH_2_PO_4_, 20 mM HEPES, 5.5 mM glucose, 5 mM NaHCO_3_, and 1 mM CaCl_2_, pH 7.4.

### ΔΨ_m_ measurements

Cells were incubated with 50 nM tetramethyl rhodamine methyl ester (TMRM; Sigma-Aldrich, T5428) in RM for 30 minutes at room temperature, before they were transferred to a Leica SP5 confocal microscope (63X objective) for imaging. TMRM is a red-fluorescing dye that accumulates within mitochondria, and its signal intensity is a function of membrane potential^21^. Instrument settings were kept constant between experiments. The TMRM fluorescence intensity of mitochondrial regions of interest were determined using the ImageJ software.

### Mitochondrial volume

MEFs were treated as indicated in the figure legends and then loaded with 50 nM TMRM and 5 µM Fluo-4 am (Life Technologies, F-23917) to visualize mitochondria and the whole cell respectively, for 40 minutes at room temperature^22^. Z-stacks were acquired using a Leica SP5 confocal microscope (63X objective). Analysis was performed using the Volocity software and the mitochondrial volume was determined as the proportion of the cytoplasm that is occupied by mitochondria.

### Mitochondrial ROS imaging

MEFs were treated as indicated in figure legends and then incubated with 2.5 µM mitoSOX (Life Technologies, M-36008) in RM for 30 minutes at 37 °C. Cells were rinsed twice in RM and then transferred to a Leica SP5 confocal microscope (63X objective) for live imaging. Instrument settings were kept constant between experiments. The mitoSOX fluorescence intensity of mitochondrial regions of interest were determined using the ImageJ software.

### Protein extraction and sub-cellular fractionation

Cells were suspended in 500 µL lysis buffer (150 mM NaCl, 1% v/v Triton X-100, 20 mM Tris pH 7.4) containing protease inhibitor cocktail (Roche, 05892791001) for 20 min on ice. Cell debris was removed by centrifugation at 12,000 g for 10 min to obtain the supernatant whole cell lysate. When sub-cellular fractionation was required, cells were suspended in 500 μL fractionation buffer (250 mM Sucrose, 10 mM KCl, 1.5 mM MgCl_2_, 1 mM EDTA, 1 mM EGTA, 20 mM HEPES, pH 7.4) containing protease inhibitor cocktail and passed through a 26-gauge needle 20 times using a 1 mL syringe, followed by 20 minutes incubation on ice. Unbroken cells and nuclei were removed by centrifugation at 800 g for 5 minutes at 4 °C. Supernatants were transferred to fresh tubes and centrifuged at 10000 g for 10 minutes at 4 °C. Subsequent supernatants were collected as the cytosolic fractions, while mitochondrial pellets were washed once in fractionation buffer and then centrifuged at 10000 g for 10 minutes at 4 °C. Finally, mitochondrial pellets were suspended in standard lysis buffer for 30 minutes on ice and the supernatant lysate was obtained after centrifugation at 10000 g for 10 minutes at 4 °C.

### Determination of cellular Oxygen Consumption Rate (OCR)

On the day before experiment, treated and untreated cells were seeded in triplicate on Seahorse XFp cell culture miniplates (3 × 10^4^ cells per well). OCR was measured with a Seahorse XFp extracellular flux analyser, using a Seahorse XFp cell mito stress test kit and following manufacturer’s instructions (as reported in Supplemental Experimental Procedures).

Data obtained from the assay were analysed with the Seahorse XFp analysis software and normalized to the amount of mitochondrial protein of each sample, in order to avoid misinterpretation of data due to differences in mitochondrial mass between samples (as explained in ref. 23).

### Western blotting

Sample proteins were quantified using a bicinchoninic acid (BCA) protein assay kit (Fisher Scientific, 13276818). Equal amounts of protein (30 µg for whole cell lysates/cytosolic fractions; 40 µg for mitochondrial fractions) were separated on 10% SDS-PAGE gels and transferred onto nitrocellulose membranes (Fisher Scientific, 10339574). The membranes were blocked in 3% non-fat dry milk in TBS-T (50 mM Tris, 150 mM NaCl, 0.05% Tween 20, pH 7.5) for 1 h and incubated overnight with the primary antibodies at 4 °C: mouse α-p62/SQSTM1 (Abcam, ab56416) 1:20000; rabbit α-ubiquitin (Abcam, ab7780) 1:1000; mouse α-β-subunit (Abcam, ab14730) 1:15000; rabbit α-PARK2 (Cell signaling, 2132 S) 1:500; mouse α-actin (Abcam, ab8226) 1:5000; mouse α-MTCO-1 (Abcam, ab14705) 1:1000. Membranes were washed in TBS-T (3 × 5 mins at RT) and then incubated with corresponding peroxidase-conjugated secondary antibodies (Dako, P0447, P0448) for 1 h at room temperature. After a further washing step (TBS-T, 3 × 5 mins at RT), proteins were detected using an ECL Plus western blotting detection kit (Fisher Scientific, 12316992). ImageJ software was used to analyze immunoreactive bands by densitometry.

### Immunofluorescence

Cells were treated as indicated in the figure legends and then fixed in 4% paraformaldehyde (PFA) for 10 mins at room temperature followed by washing with PBS (3 × 5 min). The cells were then permeabilized with 0.5% Triton-X in PBS (15 mins, RT), rinsed with PBS (3 × 5 min) and blocked with 10% goat serum and 3% bovine serum albumin in PBS for 1 h at room temperature. The cells were then incubated with primary antibodies diluted in blocking solution overnight at 4 °C. Following a further wash step, secondary antibodies were incubated for 1 h in blocking solution. Cells were rinsed in PBS and then mounted on slides with DAPI mounting medium (Abcam, ab104139). Cells were stained with the following primary antibodies: mouse α-β-subunit (Abcam, ab14730) 1:1000; α-p62/SQSTM1 (Abcam, ab56416) 1:500; α-LC3 (Abcam, ab48394) 1:75, and the following secondary antibodies: α-mouse Alexa555 (Life Technologies, A21424) 1:1000; α-rabbit Alexa488 (Life Technologies, A11008) 1:1000.

### mt-Keima analysis

Mt-Keima has an excitation spectra that is sensitive to pH changes: at neutral pH it exhibits a λ_max_ at ~440 nm, while in an acidic environment it is preferentially excited by a longer excitation wavelength of ~586 nm^24^. Following a 24 h treatment as indicated in the figure legends, Z-stacks were acquired by dual-excitation ratiometric imaging of mt-Keima transfected MEFs using a Leica SP5 confocal microscope (63X objective) equipped with 458 nm Argon gas and 543 nm Helium-Neon gas lasers. Ratiometric (543 nm/458 nm) analysis was performed using the ImageJ software and the mitophagy index was determined by dividing the total area of lysosomal (543 nm) signal to that of mitochondria (458 nm).

### Statistical analysis

All statistical analysis was performed on R software, version 3.0.2. Shapiro-Wilk tests were used to analyze the normality of data sets with N < 50. Quantile-Quantile plots were used for larger data sets. Analysis of Variance (ANOVA) and Kruskal-Wallis tests were performed accordingly to the distribution of the data sets. Multiple comparison t-tests and Wilcoxon Rank tests were performed with Holm adjustments^25^.

## Results

### SFN alters the effects of PMI on mitochondrial network content

**Figure 1:**
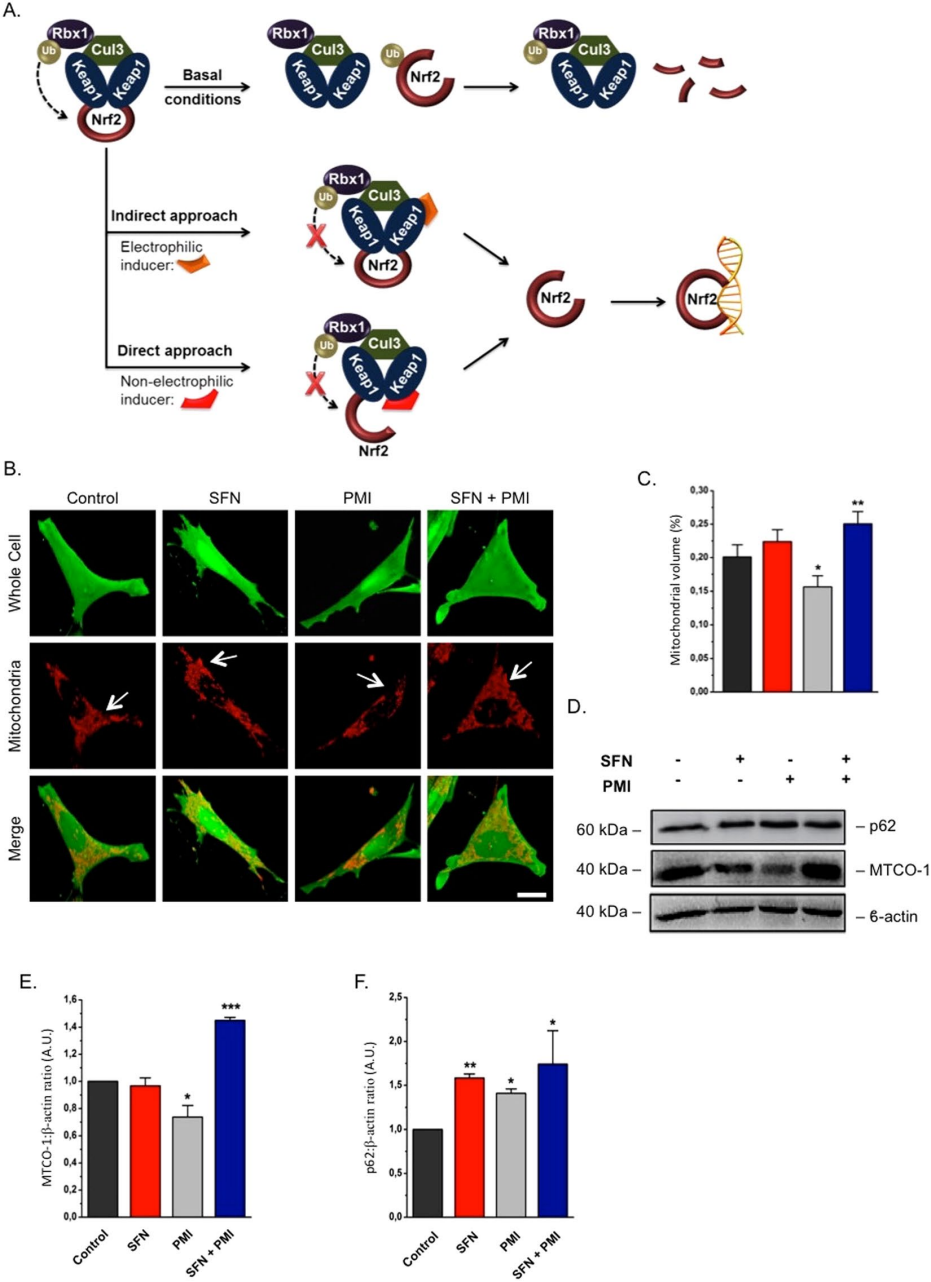
SFN halts the PMI-induced reduction of mitochondrial biomass. (**A**) Graphical representation of the Keap1-Nrf2 pathway and the different modes of chemical intervention^35^. (**B**) Representative 3-D confocal images demonstrating differences in mitochondrial mass of cells treated with DMSO vehicle control, 1 μM SFN and/or 10 μM PMI for 24 h. Cells were loaded with TMRM (red) and Fluo-4 am (green) to visualize mitochondria and the whole cell respectively. Scale bar represents 10 μm. (**C**) Quantification of mitochondrial volume, determined as the proportion of the whole cell volume occupied by the mitochondrial network (n > 20 cells; *p < 0.05, **p < 0.01) (**D**) Western blot analysis of p62 and MTCO-1 levels in MEFs treated with DMSO vehicle control, PMI (10 μM) and/or SFN (1 μM) for 24 h. β-actin is shown as a loading control. (**E** and **F**) Graphs show (**E**) MTCO-1:β-actin and (**F**) p62:β-actin ratio band density analysis (n = 3, *p < 0.05, **p < 0.01, ***p < 0.001). All values are mean ± SD.

The working model of indirect electrophilic inducer (SFN) and direct, non-electrophilic inducer (PMI) strategies to inhibit the Keap1-mediated degradation of Nrf2 is reported in Fig. 1A. Both lead to the nuclear accumulation of Nrf2 and the subsequent transcriptional activation of genes. SFN, however, does not mediate a reduction in mitochondrial protein levels similar to PMI^12^, we were therefore curious to investigate the outcome of their co-administration on the appearance of the mitochondrial network. We initially monitored the mitochondrial content of MEFs following a 24 h treatment with SFN by live imaging using the fluorescent dyes TMRM and Fluo-4 am to stain mitochondria and the whole cell respectively^22^. Cells treated with 10 µM PMI exhibited a reduced mitochondrial volume (15.66% ± 1.63 of the cell volume) contrary to control (20.10% ± 1.83) and 1 µM SFN (22.41% ± 1.75) (Fig. 1B,C). Surprisingly, addition of SFN reversed the effects of co-administered PMI and resulted in a denser mitochondrial network (25.03% ± 1.83), suggesting that the two compounds mediate antagonistic effects. This prompted us to examine the levels of the mitochondrial inner membrane protein MTCO-1 (mitochondrial Complex IV subunit 1) by immunoblotting analysis of MEFs co-treated with PMI and SFN for 24 h (Fig. 1D,E). In agreement with our previous report, PMI reduced the amount of MTCO-1 (0.74 ± 0.08) relative to control (1.00 ± 0.00), which in contrast remained unaltered in cells exposed to SFN (0.97 ± 0.06). Interestingly, the levels of MTCO-1 in cells co-treated with SFN and PMI were elevated (1.45 ± 0.02) compared to control, suggesting that SFN inhibits the mitochondrial network refinement attributed to PMI. In parallel, we monitored the levels of the Nrf2-responsive protein p62, an autophagy adaptor required by PMI to induce mitophagy. As expected, p62 concentrations, determined by immunoblotting, were up-regulated relative to controls across all treatment conditions (Fig. 1D,F; control: 1.00 ± 0.00, SFN: 1.58 ± 0.05, PMI: 1.41 ± 0.05, SFN + PMI: 1.74 ± 0.38). In support of these data, the p62 mRNA levels in cells treated with PMI and/or SFN for 24 h were elevated compared to control (Figure S1A; control: 1.00 ± 0.00, SFN: 4.38 ± 0.64, PMI: 2.00 ± 0.29, SFN + PMI: 3.65 ± 1.16).

### SFN inhibits PMI-induced mitochondrial accumulation of p62, LC3 recruitment and subsequent mitophagy

**Figure 2:**
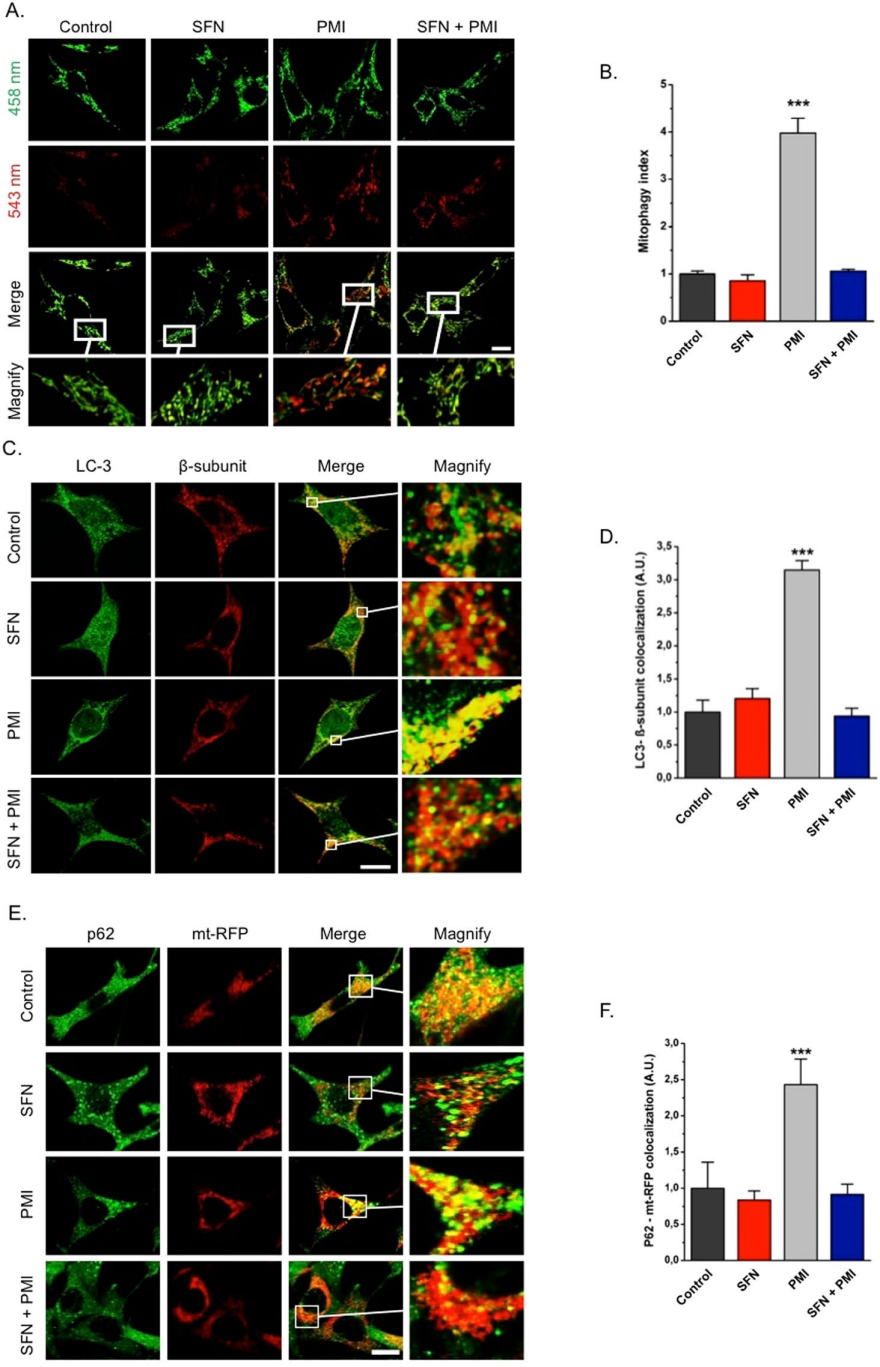
SFN inhibits the mitochondrial accumulation of LC3 and p62 in response to PMI treatment. (**A**) Representative images of MEFs transfected with mt-Keima and treated with DMSO vehicle control, 1 μM SFN and/or 10 μM PMI for 24 h. The cells were imaged with 458 nm (shown in green) or 543 nm (shown in red) light excitation. Scale bar represents 10 μm. A magnification of the merge images is shown in areas demarcated by the white box (n > 15). (**B**) Quantification of mitophagy index (n > 15, ***p < 0.001). (**C**) Representative high-resolution images of LC3 localization in MEF cells treated with DMSO vehicle control, 1 μM SFN and/or 10 μM PMI for 24 h and immunolabeled for LC3 (green) and β-subunit (red). Scale bar represents 10 μm. A magnification of the merge images is shown in areas demarcated by the white box. (**D**) Quantification of the extent of LC3:β-subunit co-localization (n > 20, ***p < 0.001). (**E**) Representative high-resolution images of mt-RFP overexpressing MEFs treated with DMSO vehicle control, 1 μM SFN and/or 10 μM PMI for 24 h and immunolabeled against p62. Scale bar represents 10 μm. A magnification of the merge images is shown in areas demarcated by the white box. (**F**) Quantification of the degree of p62:mt-RFP co-localization (n > 15, ***p < 0.001). All values are mean ± SD.

As the increased mitochondrial mass observed in MEFs co-treated with both compounds could be the outcome of impaired mitochondrial clearance, we sought to explore the ability of PMI to induce mitophagy in the presence of SFN. We transfected MEFs with the mitochondrial-targeted fluorescent probe mt-Keima and monitored its lysosomal delivery by dual-excitation (543 nm/458 nm) ratiometric imaging following treatment with PMI and SFN for 24 h. The pH-sensitive nature of mt-Keima allows the detection of lysosomal localized mitochondria that have high (543 nm/458 nm) fluorescence ratio values due to the red spectral shift occurring in the acidic lysosomal environment. The mitophagy index in cells exposed to SFN (0.85 ± 0.12) was similar to that of the DMSO control (1.00 ± 0.06), while in contrast PMI treatment (3.80 ± 0.31) resulted in increased lysosomal delivery of mitochondria, as indicated by their strong fluorescence at 543 nm (Fig. 2A,B). Notably, this effect was abolished in cells exposed to both compounds (1.05 ± 0.04), indicating that SFN interferes with the mitophagic pathway exploited by PMI. Further evidence of the inhibitory effect of SFN on mitophagy was obtained by immunofluorescence analysis of the degree of co-localization between the autophagosomal-localized protein LC3 and mitochondria (Fig. 2C,D). Following treatment with 10 μM PMI for 24 h, increased formation of mitochondria-containing autophagosomes (3.15 ± 0.14) was observed compared to untreated conditions (1.00 ± 0.18). Additionally, in agreement with our previous results, exposure to 1 µM SFN, either alone (1.21 ± 0.14) or in combination with 10 µM PMI (0.93 ± 0.12) for 24 h, did not initiate a mitophagic response in MEFs, as indicated by the limited degree of mitochondrial LC3 co-localization, further supporting the distinction between the two compounds.

The extended association of p62 with the mitochondrial network appears to play an important role in the activation of mitophagy by PMI^12^. Thus, the inability of SFN to activate the process, despite up-regulating p62 (Fig. 1), could be the outcome of an impaired mitochondrial localization of the autophagy receptor. To investigate this, MEFs transfected with mitochondria-targeted red fluorescent protein (mt-RFP) were treated with SFN and/or PMI for 24 h and immunolabelled for p62. High-resolution confocal images revealed a trend that is consistent with our previous observations (Fig. 2E,F): PMI triggered an extensive mitochondrial localization of p62 (2.43 ± 0.35) contrary to control (1.00 ± 0.36) and SFN (0.84 ± 0.13), which was normalized in the presence of both compounds (0.91 ± 0.15), indicating that the inhibitory effects of SFN on mitophagy may stem from the reduced association of p62 with the mitochondrial network.

Enhanced poly-ubiquitination of the outer mitochondrial membrane (OMM) proteins serves as recruiting signal for p62 translocation during PMI-induced mitophagy^12^. To investigate further the mechanism by which SFN diminishes the PMI-mediated mitophagic response, we examined the ubiquitination status of mitochondrial fractions isolated from treated MEFs. Mitochondrial poly-ubiquitination in cells treated with PMI (1.53 ± 0.29) was significantly elevated, particularly at heavier band densities, compared to control (1.00 ± 0.00) and SFN (0.92 ± 0.26) (Figure S1B and C). Notably, this effect was abolished in the presence of both compounds (0.85 ± 0.44), implying that the diminished mitochondrial recruitment of p62 and LC3 with SFN may be a consequence of the reduced ubiquitination of mitochondrial surface proteins.

### SFN impairs PARK2 translocation on polarized mitochondria

**Figure 3:**
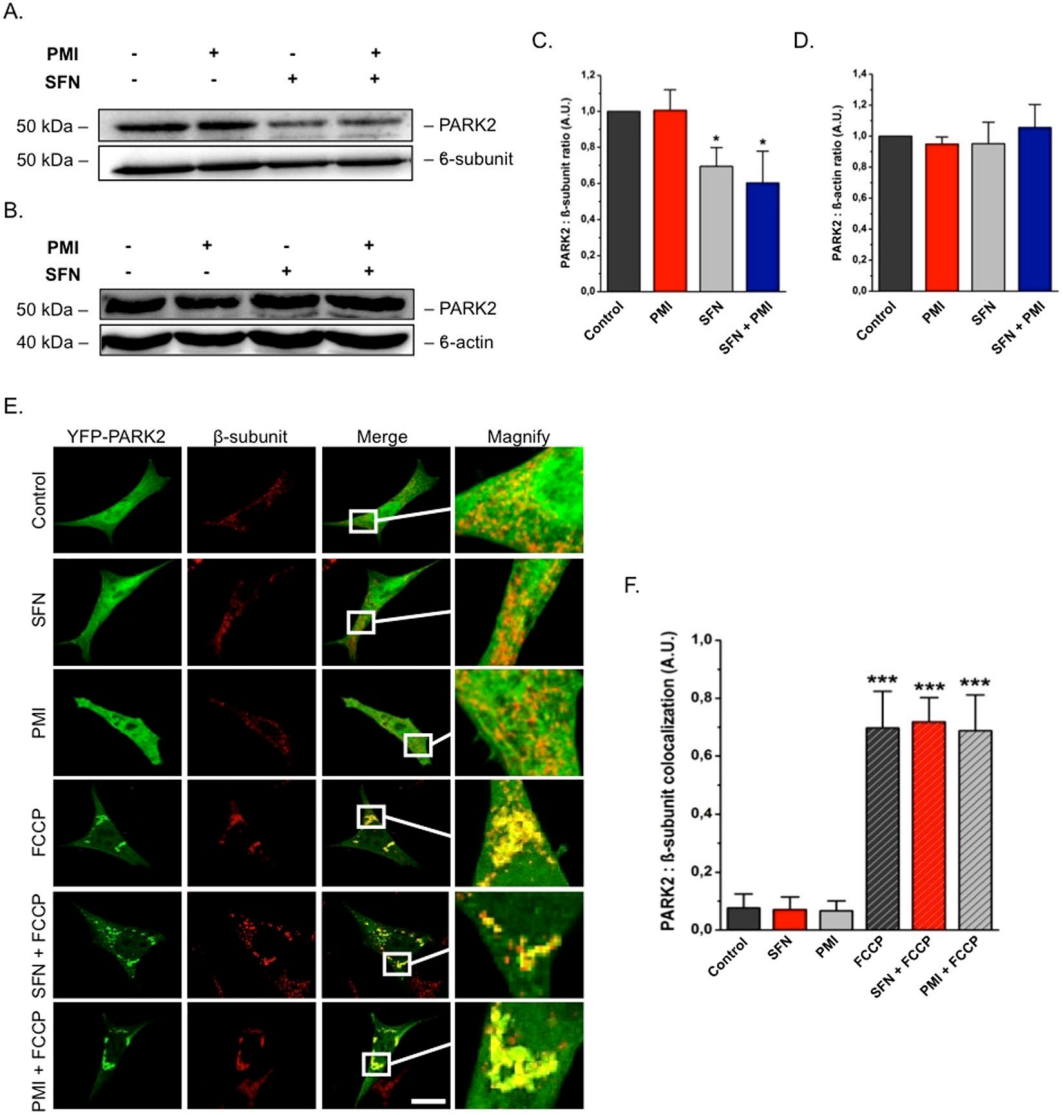
SFN reduces basal, but not FCCP-induced, PARK2 translocation. (**A**) and (**B**) Western blot analysis of PARK2 levels in (**A**) mitochondrial and (**B**) cytosolic fractions of MEF cells treated with DMSO vehicle control, 1 μM SFN and/or 10 μM PMI for 24 h. β-subunit and β-actin are shown as loading controls. (**C** and **D**) Quantification of (**C**) PARK2:β-subunit and (**D**) PARK2:β-actin ratio band density analysis (n = 3, *p < 0.05). (**E**) Representative images of PARK2 localization in MEF cells treated with DMSO vehicle control, 1 μM SFN and/or 10 μM PMI for 24 h, before and after treatment with FCCP (20 μM) for 4 h. Scale bar represents 10 μm. A magnification of the merge images is shown in areas demarcated by the white box. (**F**) Quantification of the degree of PARK2:β-subunit co-localization (n > 20, ***p < 0.001). All values are mean ± SD.

During PINK1-PARK2 driven mitophagy, PARK2 translocates to mitochondria and amplifies the signal originally generated by PINK1 by attaching ubiquitin chains on flagged mitochondria^3^. To explore the effect of SFN on this process, we monitored the localization of endogenous PARK2 by western blotting analysis of isolated mitochondrial (Fig. 3A,C) and relative cytosolic counterparts (Fig. 3B,D) derived from treated MEFs. PMI exposure (24 h) did not affect the relocation of PARK2 to mitochondria (1.04 ± 0.09) relative to control (1.00 ± 0.00). However, a reduced association of the E3 ligase with the mitochondrial network was observed in the presence of SFN (0.70 ± 0.10), an effect that was not reversed upon PMI addition (0.53 ± 0.12). Notably, the cytosolic amounts of PARK2 remained unchanged throughout all conditions (Fig. 3B,D), indicating that Nrf2 induction does not alter its expression levels (control: 1.00 ± 0.00, PMI: 0.95 ± 0.05, SFN: 0.95 ± 0.14, SFN + PMI: 1.06 ± 0.15).

The decreased mitochondrial association of PARK2 in the presence of SFN could result from an inhibition of its translocation ability^26^. To investigate this, we monitored the cellular localization of YFP-PARK2 under FCCP-induced mitochondrial stress via confocal imaging of treated MEFs immunolabeled against β-subunit. Under resting physiology, YFP-PARK2 was uniformly distributed within cells throughout all treatment conditions (Fig. 3E,F; control: 0.08 ± 0.05, SFN: 0.07 ± 0.04, PMI: 0.07 ± 0.03). However, challenging cells with the protonophore FCCP triggered a redistribution and extended mitochondrial localization of YFP-PARK2, which remained unaltered in the presence of SFN or PMI (Fig. 3E,F), suggesting that its ability to translocate to damaged mitochondria is not compromised by either compound (control + FCCP: 0.70 ± 0.13, SFN + FCCP: 0.72 ± 0.08, PMI + FCCP: 0.69 ± 0.12).

### Reversible, but not irreversible, inhibitors of Keap1 activate mitophagy

**Figure 4:**
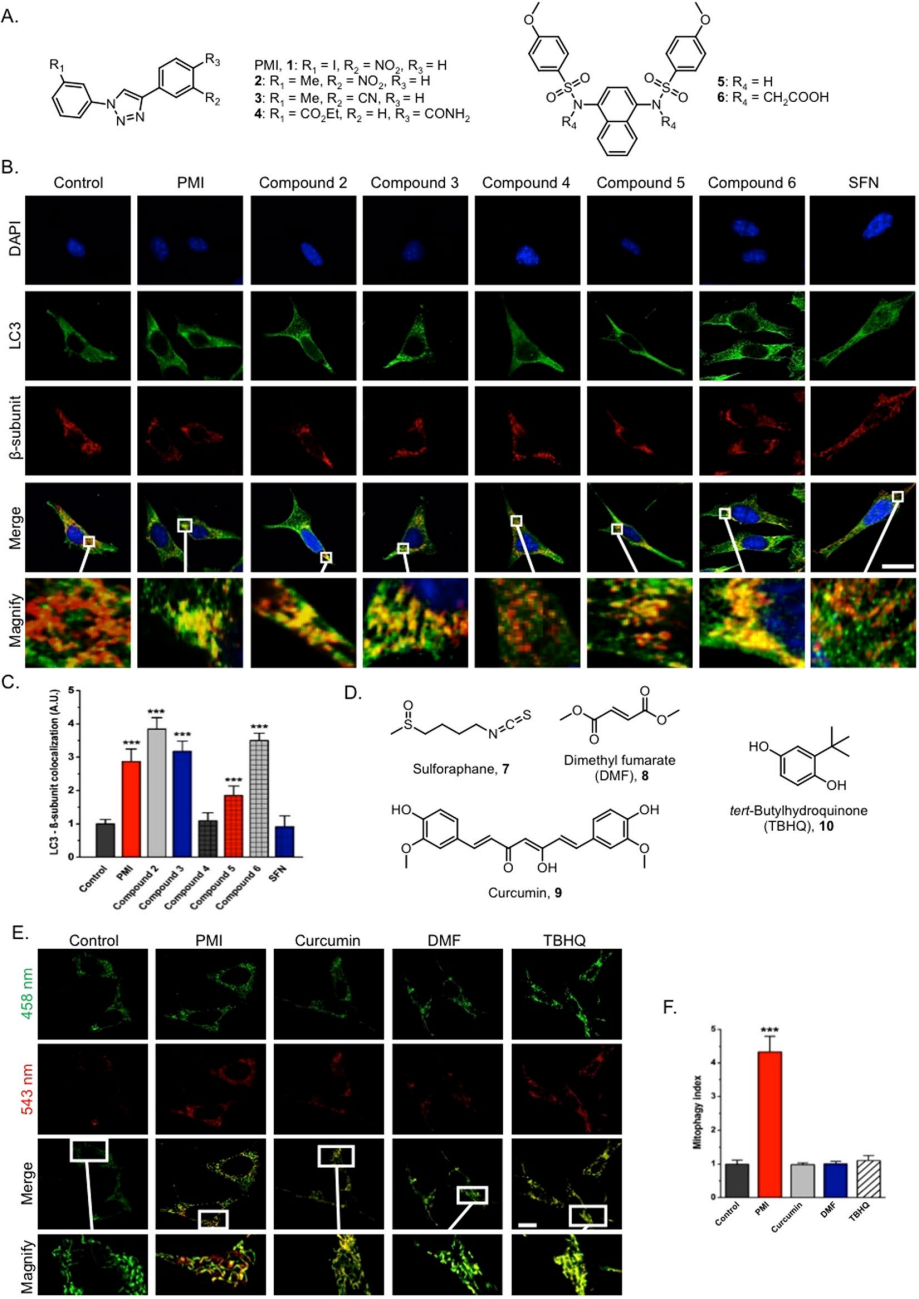
Reversible, but not irreversible, Keap1 inhibitors promote mitophagy. (**A**) Chemical structures of reversible Keap1 inhibitors used in this study. (**B**) Representative high-resolution images of LC3 localization in MEF cells treated with DMSO vehicle control, 1 μM SFN or compounds **1**–**6** (10 μM **1**–**4** and **6**, 30 μM **5**) for 24 h and immunolabeled for LC3 (green) and β-subunit (red). DAPI (blue) is representative of cell nuclei. Scale bar represents 10 μm. A magnification of the merge images is shown in areas demarcated by the white box. (**C**) Quantification of the extent of LC3:β-subunit co-localization (n > 20, ***p < 0.001). (**D**) Chemical structures of irreversible Keap1 inhibitors used in this study. (**E**) Representative images of MEFs transfected with mt-Keima and treated with DMSO vehicle control, PMI (10 μM) or compounds **8**–**10** (40 μM DMF, 5 μM curcumin, 20 μM TBHQ) for 24 h. The cells were imaged with 458 nm (shown in green) or 543 nm (shown in red) light excitation. Scale bar represents 10 μm. A magnification of the merge images is shown in areas demarcated by the white box (n > 20). (**F**) Quantification of mitophagy index (n > 15, ***p < 0.001). All values are mean ± SD.

The potential of other Keap1-Nrf2 PPI inhibitors to trigger mitochondrial clearance was then investigated. To do so, we selected a small number of PMI analogues in order to explore the structure-activity relationship (SAR) of this chemical series (Fig. 4A: compounds 1–4) and compared them to two recently described PPI inhibitors that are structurally distinct to PMI (Fig. 4A: compounds 5–6)^18, 19^. To assess their effects on mitophagy, we monitored the recruitment of LC3 to mitochondria by immunofluorescence analysis of treated cells. Exposure to SFN did not alter the mitochondrial association of LC3 relative to control, while on the contrary an extensive autophagosomal localization of mitochondria was recorded throughout all other treatment conditions (Fig. 4B,C). In particular, the PMI derivative **2** and the naphthalene compound **6** were the most active inducers amongst the compounds screened, while treatment with **5** resulted in a relatively reduced mitochondrial LC3 localization, which is in line with its reduced Nrf2-inducing potential (control: 1.00 ± 0.13, PMI (**1**): 2.87 ± 0.38, compound **2**: 3.84 ± 0.35, compound **3**: 3.18 ± 0.30, compound **4**: 1.09 ± 0.24, compound **5**: 1.85 ± 0.29, compound **6**: 3.50 ± 0.22, SFN (**7**): 0.91 ± 0.34)^18^. Interestingly, the PMI analogue **4**, which served as a negative control in our experiments due to the lack of Nrf2-inducing activity^13^, did not trigger mitophagy, further supporting the proposed Nrf2-dependent mechanism. Further indications of the Nrf2-dependence of the activity were obtained testing the KEAP1^−/−^ MEFs^27^ in which the effect of the compound elapsed whilst an underlying level of mitophagy was instead detectable (Figure S2A,B).

Having shown that in contrast to SFN, reversible Keap1 inhibitors induce mitophagy, we were curious to explore the potential of other well-studied electrophilic Nrf2 inducers to generate a similar response. Given the differential time-dependent effects of such compounds on Nrf2 stabilization, mt-Keima was chosen as a readout due to its resistance to lysosomal degradation, which allows the cumulative detection of mitophagy^24, 28^. Dimethyl fumarate (DMF, **8**), curcumin (**9**) and *tert*-butylhydroquinone (TBHQ, **10**) were screened for their ability to promote mitochondrial autophagy in mt-Keima transfected MEFs (Fig. 4D,E,F). Following treatment with compounds **8**–**10** for 24 h, no apparent effect on mitophagy was detected compared to control, while consistently with its robust mitophagy-inducing properties, PMI gave a positive result (control: 1.00 ± 0.11, PMI: 4.33 ± 0.46, curcumin: 0.98 ± 0.05, DMF: 1.00 ± 0.08, TBHQ: 1.11 ± 0.15). Collectively, these data suggest that in sharp contrast to direct inhibitors of the Keap1-Nrf2 PPI, electrophilic Nrf2 inducers do not promote mitochondrial autophagy.

### PMI mediates a more pronounced improvement in superoxide metabolism compared to SFN

**Figure 5:**
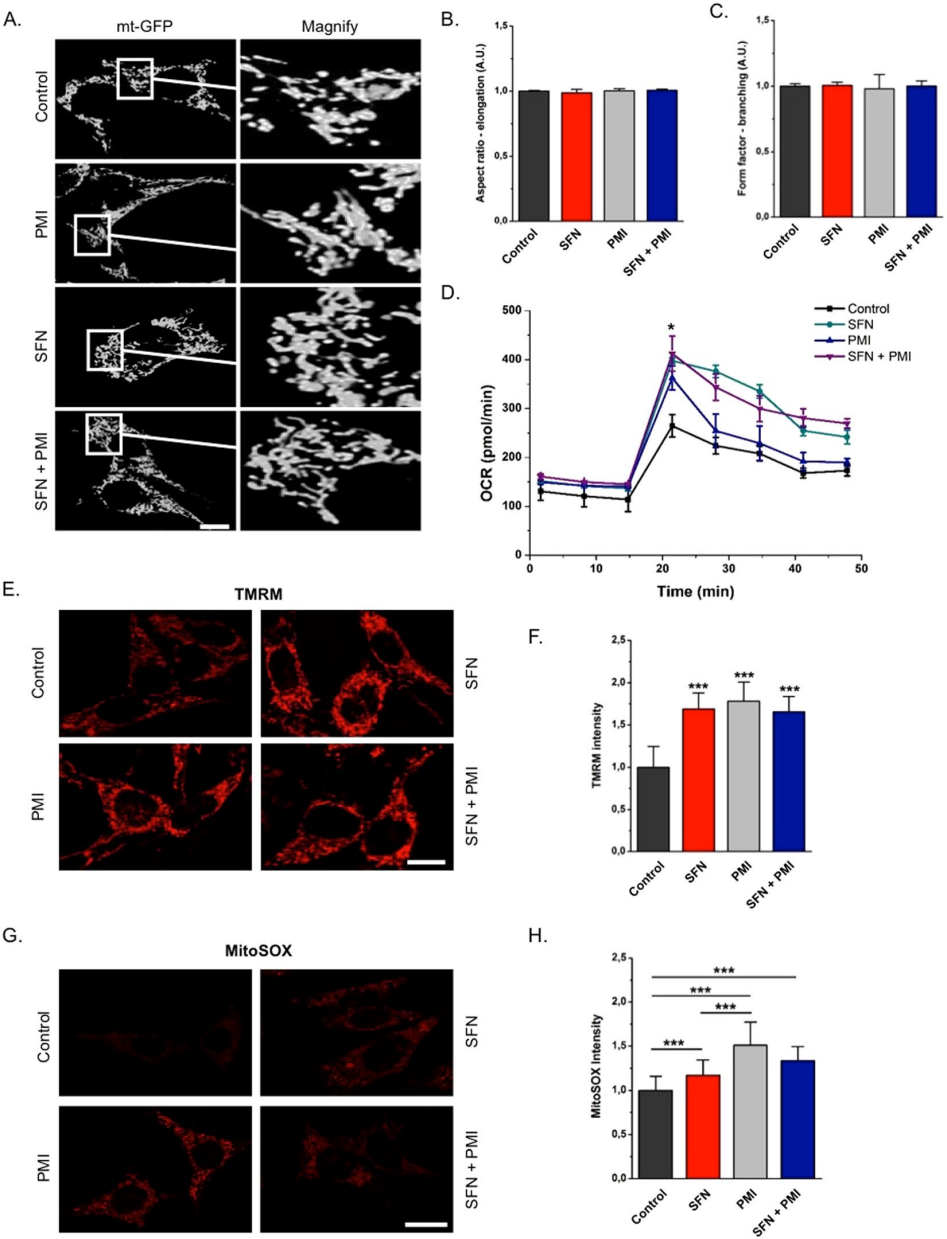
Oxidative metabolism within mitochondria is more enhanced in the presence of PMI compared to SFN. (**A**) Representative high-resolution confocal images of MEF cells transfected with mt-GFP and treated with DMSO vehicle control, 10 μM PMI and/or 1 μM SFN for 24 h. Scale bar represents 10 μm. A magnification of the merge images is shown in areas demarcated by the white box. (**B** and **C**) Graphs showing no differences throughout conditions in (**B**) elongation and (**C**) branching of mitochondria (n > 15). (**D**) Graph showing OCR of cells treated with DMSO vehicle control, 10 μM PMI and/or 1 μM SFN for 24 h, n ≥ 2. (**E**) Representative high-resolution confocal images showing differences in basal ΔΨ_m_ of MEFs treated with DMSO vehicle control, 10 μM PMI and/or 1 μM SFN for 24 h and loaded with the potentiometric fluorescent probe TMRM (red) for 30 min. Scale bar represents 10 µm. (**F**) Quantification of mean TMRM fluorescence intensity (n > 30, ***p < 0.001). (**G**) Representative confocal images of MEFs treated with DMSO vehicle control, 10 μM PMI and/or 1 μM SFN for 24 h and incubated with the mitochondrial superoxide sensitive probe mitoSOX (red) for 30 min. (**H**) Quantification of mean mitoSOX fluorescence intensity (n > 40, ***p < 0.001). All values are mean ± SD.

Having demonstrated the contrasting effects of PPI inhibitors and electrophilic inducers on mitochondrial volume (Fig. 1) and clearance (Figs 2 and 4), we were curious to examine their effects on aspects of mitochondrial morphology and function. Induction of mitophagy is often accompanied by fragmented mitochondria, while an uncontrolled sequestration of organelles could result in a disorganized appearance of the network^29, 30^. Analysis of high-resolution confocal images of mt-GFP overexpressing MEFs revealed no significant differences in connectivity and shape of mitochondria across all treatment conditions, suggesting that mitochondrial fragmentation is not a prerequisite for PMI-induced mitophagy and that the connectivity of the network is not altered in response to the inhibitory activity of SFN on mitophagy (Fig. 5A,B,C; Aspect ratio - control: 1.00 ± 0.01, SFN: 0.99 ± 0.03, PMI: 1.00 ± 0.02, SFN + PMI: 1.01 ± 0.01; Form factor - control: 1.00 ± 0.02, SFN: 1.01 ± 0.02, PMI: 0.98 ± 0.11, SFN + PMI: 1.00 ± 0.04).

To investigate the effects of PMI and SFN on mitochondrial respiration we analyzed the oxygen consumption rate (OCR) of cells treated with the above compounds for 24 h. The maximal OCR rate, induced by the co-administration of FCCP and oligomycin, was enhanced throughout all treatment conditions relative to control (Fig. 5D; pmol/min units control: 264.52 ± 22.59, SFN: 397.66 ± 6.10, PMI: 362.56 ± 24.34, SFN + PMI: 412.19 ± 35.85). It is interesting to note that despite the reduced mitochondrial mass of cells treated with PMI (Fig. 1), the extent of their maximal oxygen consumption is similar to that of cells exposed to SFN, either alone or in combination with PMI, suggesting an enhanced respiratory capacity.

Mitochondrial membrane potential (ΔΨ_m_), an important read-out of mitochondrial health, is augmented in cells exposed to PMI^12^. To investigate whether SFN has a similar effect, MEFs treated with PMI and SFN for 24 h, were loaded with the potentiometric dye TMRM, and monitored using high-resolution confocal imaging. Increased ΔΨ_m_ was detected relative to control across all treatment conditions, arguing for a mitophagy-independent effect of Nrf2 inducers on basal ΔΨ_m_ levels (Fig. 5E,F; control: 1.00 ± 0.25, SFN: 1.69 ± 0.19, PMI: 1.78 ± 0.22, SFN + PMI: 1.66 ± 0.18).

We then sought to explore whether the improved mitochondrial respiratory activity in cells treated with PMI or SFN could lead to a greater superoxide generation within the organelle. To do so, we incubated MEFs with the fluorescence probe mitoSOX, which allows the selective detection of mitochondrial superoxide radicals, and monitored its oxidation by live imaging analysis (Fig. 5G,H). Exposure to SFN, either alone (1.16 ± 0.17) or in presence of PMI (1.34 ± 0.16), led to a moderate increase in the mitoSOX intensity relative to control (1.00 ± 0.16). Intriguingly, we observed a statistical significant difference in the levels of mitochondrial superoxide generation between PMI (1.51 ± 0.26) and SFN (1.16 ± 0.17) treatments, which may account for the enhanced oxidative metabolism consistent with the OCR data obtained (Fig. 5D). As above, KEAP1^−/−^ MEFs were also tested for the mitochondrial levels of superoxide, which were increased even in the absence of PMI (Figure S2C), consistent with constitutive Nrf2 activation. This underlined greater Nrf2 activity thus resulting in cleaning up of the mitochondrial network and improvement of its performance.

### Mitochondrial superoxide generation plays a crucial role in PMI-induced mitophagy

**Figure 6:**
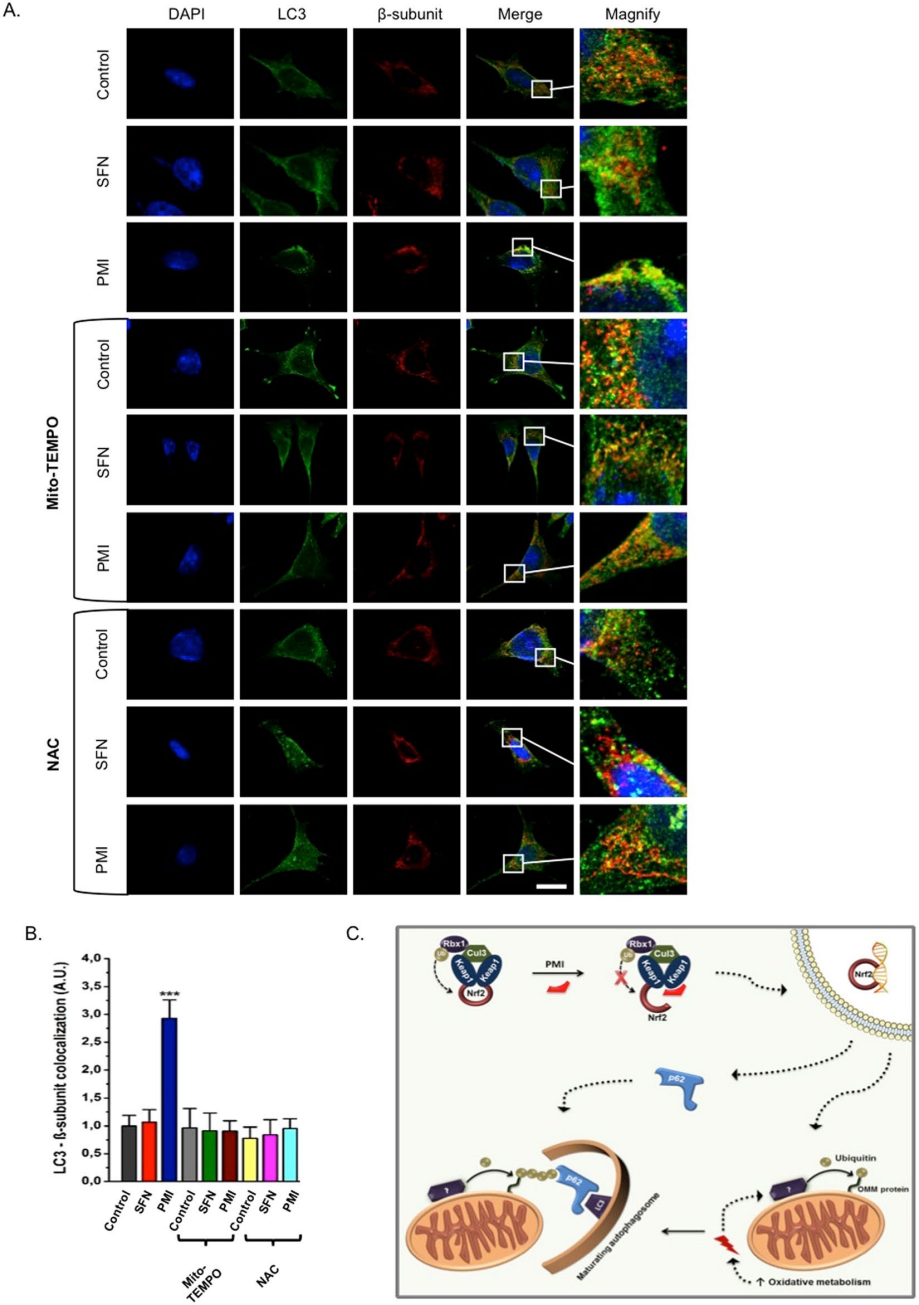
Selective scavenging of mitochondrial superoxides inhibits the PMI-mediated initiation of mitophagy. (**A**) Representative high-resolution images of LC3 localization in MEF cells treated with DMSO vehicle control, 1 μM SFN, or 10 μM PMI for 24 h in the presence or absence of 50 µM mito-TEMPO or 5 mM NAC. Cells were immunolabeled for LC3 (green) and β-subunit (red). Scale bar represents 10 μm. A magnification of the merge images is shown in areas demarcated by the white box. (**B**) Quantification of the extent of LC3:β-subunit co-localization (n > 10, ***p < 0.001). (**C**) Inhibition of the PPI between Keap1 and Nrf2 leads to the nuclear accumulation of the latter and the subsequent transcriptional activation of genes involved in the regulation of mitochondrial function and autophagy, including p62. As a result, the rate of oxidative metabolism is increased and the mitochondrial redox status is altered, which initiates a mitophagic response that targets mitochondria to autophagosomes via p62.

Given the involvement of ROS in the regulation of mitophagy^31–33^, we next sought to explore whether the mitophagic activity of PMI is sensitive to alterations in mitochondrial superoxide levels. We monitored the delivery of mitochondria to autophagosomes in PMI- or SFN-treated MEFs in the presence of Mito-TEMPO, a mitochondria-targeted superoxide specific scavenger^34^. LC3 localization analysis revealed a complete inhibition of PMI-mediated mitophagy by mito-TEMPO, suggesting that mitochondrial superoxide production plays an important role in the mitophagic cascade exploited by PMI (Fig. 6A,B; control: 1.00 ± 0.19, SFN: 1.07 ± 0.22, PMI: 2.93 ± 0.33, control + Mito-TEMPO: 0.97 ± 0.34, SFN + Mito-TEMPO: 0.91 ± 0.32, PMI + Mito-TEMPO: 0.90 ± 0.19). Interestingly, non-selective ROS scavengers, such as the glutathione precursor *N*-acetylcysteine (NAC) affect the autophagosomal localization of mitochondria in PMI-treated cells in a similar fashion, whilst no differences were observed in SFN-treated cells (Fig. 6A,B; control + NAC: 0.78 ± 0.20, SFN + NAC: 0.84 ± 0.27, PMI + NAC: 0.95 ± 0.18).

A global analysis of mitophagy receptor expression levels in SH-SY-5Y cells treated with PMI, SFN or the combination showed no significant modifications in the mRNA levels of any of these proteins^35^ (Figure S2D). Collectively, these results are consistent with the mitophagic activity of PMI being influenced by the mitochondrial redox status as depicted in Fig. 6C.

## Discussion

Efforts to pharmacologically modulate the process of mitohagy have been rather limited despite the recent progress in understanding its underlying molecular mecchanisms.^3, 28, 36^. The vast majority of mitophagy inducers are mito-toxins^37^, which *per se* restricts their further development into therapeutic agents and highlights the need for alternative chemical tools to activate and/or modulate the process.

We previously described the characterization of the novel Keap1 inhibitor HB229/PMI as an inducer of mitophagy that does not depolarize the ΔΨ_m_ or cause any apparent toxicity to the organelle^12^. Intriguingly, our preliminary results suggested that the electrophilic Nrf2 inducer SFN may have alternative effects on mitochondria, which we investigated further.

Here we report that unlike PMI, SFN does not stimulate mitophagy (Figs 1 and 2) even though it does exploit the identycal Nrf2 pwthay upregulating p62 (Fig. 1D,F and G). Instead, it impedes the PMI-mediated initiation of mitophagy by restricting mitochondrial ubiquitination and preventing the subsequent recruitment of p62 (Fig. 3, S1A and S1B), an indispensable event in the mitophagic cascade exploited by PMI^12^. The increased size of the mitochondrial network observed in co-treated cells (Fig. 1) further supports this and suggests that in the presence of both compounds, the effects of SFN are likely predominant and the Nrf2-mediated coordination between mitochondrial autophagy and biogenesis is disrupted favouring the accumulation of the organelles.

Similarly to SFN, the electrophilic Nrf2 activators TBHQ, DMF and curcumin do not stimulate mitophagy, whereas selected analogues from two structurally distinct classes of Keap1-Nrf2 PPI inhibitors induce extended autophagosomal localization onto mitochondria (Fig. 4). This is evidence that the biological effects of reversible Nrf2 inducers on mitochondrial quality control are different from t﻿hose mediated by the electrophilic Nrf2 inducers.

Despite their differential effects on mitophagy, PMI and SFN exert similar effects on mitochondrial morphology and the bioenergetic capacity of treated cells (Fig. 5). These results are consistent with the increased mitochondrial respiration observed in both Keap1 KO and Keap1 KD cells^22, 38^. Nrf2 appears to influence mitochondrial respiration by regulating the availability of substrates (NADH, FADH_2_ and succinate)^22^, which may explain the elevated ΔΨ_m_ and superoxide generation observed in the presence of SFN and PMI.

Our results suggest that the effects of PMI on mitochondrial superoxide metabolism are more pronounced compared to SFN (Fig. 5F,G) – however, it is possible that the two compounds exert their effects over a different time course, or with a different dose-response profile. Nonetheless, the inhibition of mitophagy by mito-TEMPO is intriguing and suggests a possible role for mitochondrial superoxide metabolism in the mitophagic cascade exploited by PMI (Fig. 6A,B). Similarly to mito-TEMPO, NAC supplementation suppressed the mitophagic activity of PMI, further supporting our hypothesis. Indeed, several studies suggest an involvement of ROS in the regulation of OMM ubiquitination mechanisms^29, 39^, which may control the recruitment of p62 during PMI-mediated mitophagy.

In our working model (Fig. 7) we therefore propose that by inhibiting Keap1, PMI promotes an Nrf2-mediated improvement in mitochondrial health and function. The activation of mitophagy by PMI may thus act as a preventive mechanism that recycles aged and damaged mitochondria from cells, thereby renewing the pool of the healthy ones to maintain efficient respiratory activity.

In support of this hypothesis, subtle increases in mitochondrial ROS levels are known to induce a mitochondria-targeted autophagic response^40^, which resembles the selective activation of mitophagy induced by PMI, but not general autophagy^12^. Moreover, increased mitochondrial respiration has been previously shown to activate mitophagy through recruitment of the GTPase Rheb to mitochondria^41^. A respiration-dependent mechanism could also explain the diminished mitophagy in cells co-treated with PMI and the mitochondrial uncoupler FCCP^12^. Interestingly, SKN-1, the nematode homologue of Nrf2, plays a similar role in mitochondrial homeostasis in *Caenorhabditis elegans* and activates mitophagy in response to mitochondrial dysfunction and oxidative stress^42^.

Although further work is required to elucidate the exact mechanism responsible for the differential effects observed, it is possible that different modes of Keap1 inhibition may lead to distinct biological profiles. PMI does appear to promote an ‘open’ Keap1-Nrf2 complex, whereas SFN and other electrophilic inducers result in a ‘closed’ inactive complex (Fig. 1A)^13, 43^ besides mediating a number of off-targets effects such as the activation of autophagy. It is possible that the differing Keap1 inhibition mechanisms affects the interaction with other Keap1 binding partners in different ways. Of note is without doubts the interplay between Keap1 and p62, with the latter being both upregulated by and in competition with Nrf2 to bind Keap1 Kelch domain as reported in the p62/SQSTM1-Keap1-Nrf2 axis:^44, 45^ a positive feedback mechanism exploited in pathophysyiological conditions^46^. Previously, we have shown that PMI promotes a targeted autophagic degradation of mitochondria without affecting general autophagy^12^, which could be instead induced by a variety of covalent Keap1 inhibitors, including those used in the current study^16, 47–49^. Both PMI and sulforaphane increase p62 concentrations in cells but the mechanistic interplay between the compounds and p62 in disrupting the Keap1-Nrf2 interaction, as well as the negative regulators of the p62 inhibition of Keap1 (e.g. TRIM21)^50^ remain to be elucidated.

It is therefore plausible that due to their reactivity, electrophilic Keap1 inhibitors may interfere with redox-sensitive pathways that regulate mitophagy, thereby masking the Nrf2-mediated effects on this process. For example, SFN has been shown to inhibit histone deacetylase 6 (HDAC-6)^51, 52^, a p62-interacting protein involved in mitophagy^53, 54^. HDAC-6 has been shown to regulate the activity of PARK2 via a direct interaction^55^, which could possibly explain the reduced association of PARK2 with the mitochondrial network in the presence of SFN (Fig. 3). SFN and other electrophiles are also capable of acting as superoxide scavengers and may therefore disrupt the subtle mitochondrial redox balance required for the initiation of mitophagy^56–58^.

The experiments presented here elaborate upon the intriguing activity of Nrf2 inducers as pharmacological probes in the area of mitophagy and indicate that non-electrophilic compounds may be amenable to therapeutic exploitation. The data suggest an anterograde-like mechanism of mitochondrial quality control that appears to be relatively free from acute toxicity. Compounds that demonstrate this type of biological activity may be instrumental in dissecting the homeostatic role of the mitophagic process in the absence of an acute, artificially triggered collapse of organelle bio-energetics.

## Electronic supplementary material

Supplementary Figures
