# Supplementary material for: Reversible Keap1 inhibitors are preferential pharmacological tools to modulate cellular mitophagy
Source: Sci Rep. 2017 Sep 4;7:10303. doi: 10.1038/s41598-017-07679-7 (PMC5583253; doi:10.1038/s41598-017-07679-7)
Supplement: Supplementary file 1 — Supplementary Figures [file 41598_2017_7679_MOESM1_ESM.pdf]

Reversible Keap1 inhibitors are preferential pharmacological tools to modulate cellular mitophagy

Nikolaos D. Georgakopoulos[1,3], Michele Frison[1], Maria Soledad Alvarez[1], Hélène Bertrand[3], Geoff Wells[3] and Michelangelo Campanella[1,2]

[1]*Department of Comparative Biomedical Sciences, The Royal Veterinary College, University of London, Royal College Street NW1 0TU, London, United Kingdom*

[2]*University College London Consortium for Mitochondrial Research, Gower Street, WC1 6BT, London, United Kingdom*

[3]*UCL School of Pharmacy, 29/39 Brunswick Square, London, United Kingdom*

To whom correspondence should be addressed:

Dr. Michelangelo Campanella

Email: mcampanella@rvc.ac.uk

or

Dr. Geoff Wells

Email: g.wells@ucl.ac.uk

Supplementary Figure 1

A.

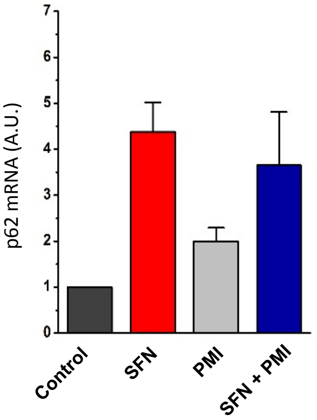

B.

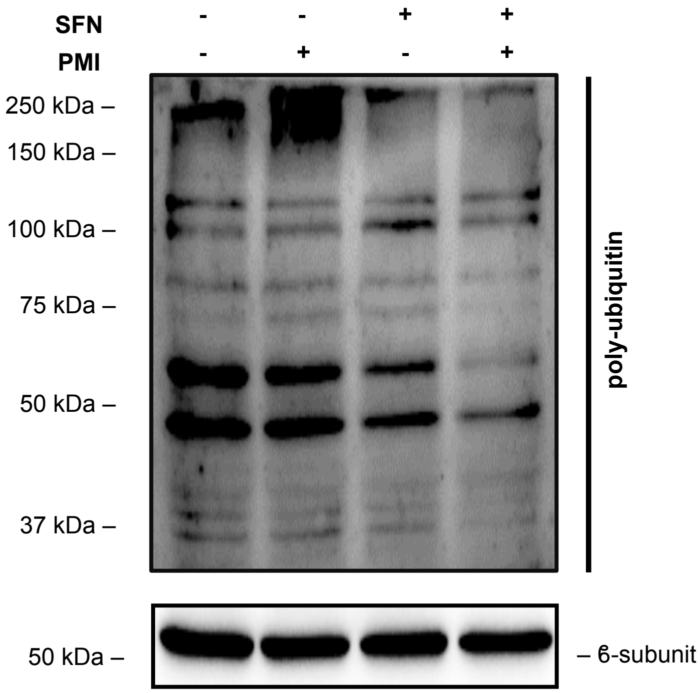

C.

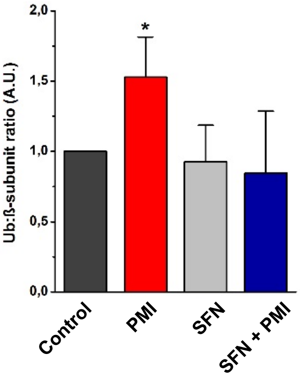

Supplementary Figure 2

A.

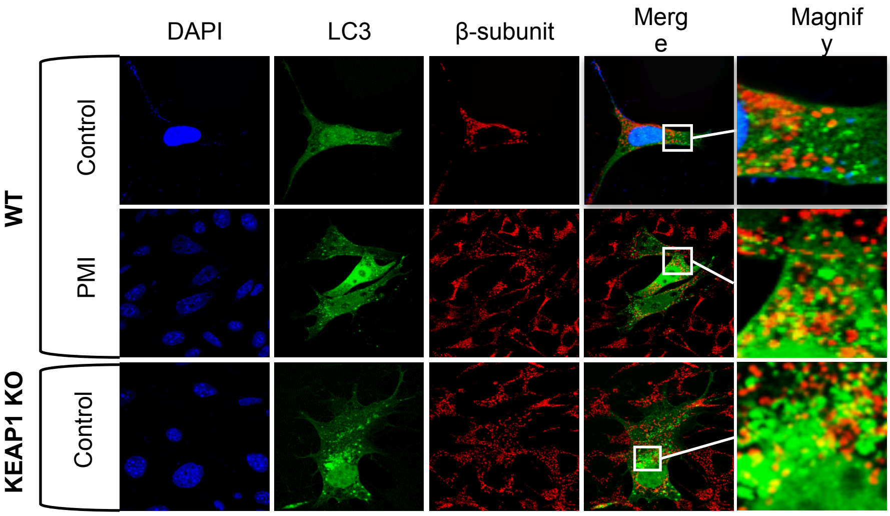

B.

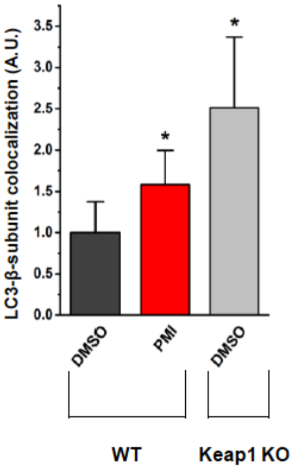

C.

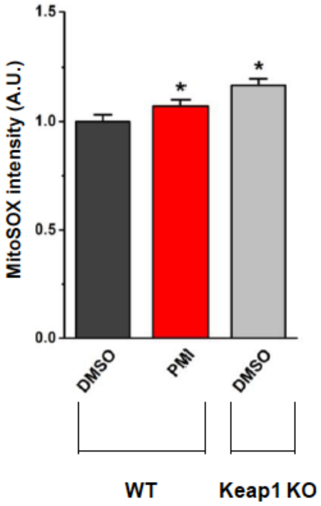

D.

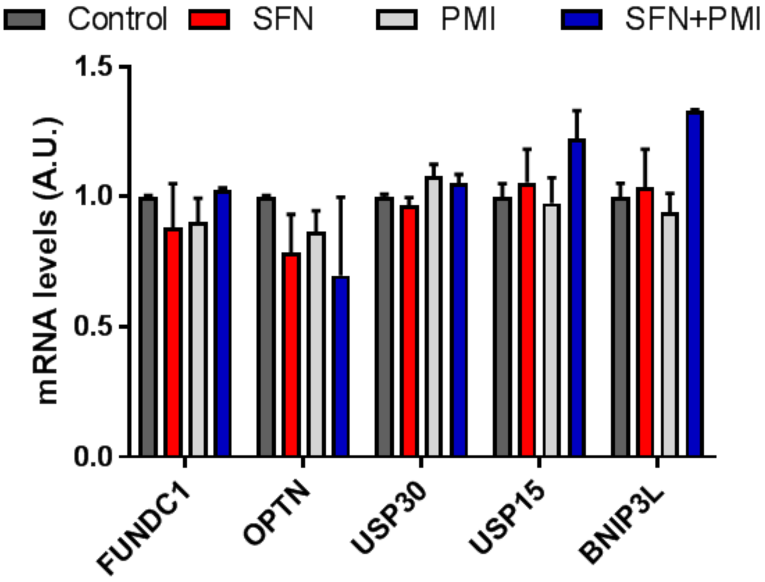

Supplementary Figure 1. Differential effects of PMI and SFN on mitochondrial ubiquitination

(A) Graph showing p62 mRNA levels in MEFs treated with DMSO vehicle control, PMI (10 μM) and/or SFN (1 μM) for 24 h, n =3. (B) Western blot of mitochondrial fractions, highlighting mitochondrial ubiquitination following 24 h treatments with DMSO vehicle control, PMI (10 μM) and/or SFN (1 μM). β-subunit is shown as loading control. (C) Graph showr Ub:β-subunit ratio band density analysis (n=3, *$p < 0.05$). All values are mean ± SD.

Supplementary Figure 2. Knockout of Keap1 is sufficient to increase mitochondrial superoxide production and induce mitophagy.

(A) Representative high resolution images of LC3 localisation in WT and KEAP1 KO MEF cells, treated with DMSO vehicle control or 10 μM PMI for 24 h. Cells were immunolabeled for LC3 (green) and β-subunit (red). Scale bar represents 10 μm. A magnification of the merge images is shown in areas demarcated by the white box. (B) Quantification of the extent of LC3:β-subunit co-localization (n > 20, *$p < 0.05$). (C) Quantification of mean mitoSOX fluorescence intensity in WT and KEAP1 KO MEF cells, treated with DMSO vehicle control or 10 μM PMI for 24 h (n > 20, ***$p < 0.001$). (D) Graph showing absence of alterations in mRNA levels of 5 mitophagy receptors in SH-SY5Y cells treated with DMSO vehicle control, 1 μM SFN, or 10 μM PMI for 8 h.
